# Fluid Assessment Scoring Tool (FAST): Development of a Novel Fluid Assessment Scoring Tool for Critically Ill Pediatric Patients—A Pilot Study

**DOI:** 10.7759/cureus.92067

**Published:** 2025-09-11

**Authors:** Mohammed Salameh, Pranali Awadhare, Jennifer Joiner, Michael Scheurer, Utpal Bhalala

**Affiliations:** 1 Critical Care Medicine, University of Texas Southwestern Medical Center, Dallas, USA; 2 Pediatrics and Child Health, Driscoll Children's Hospital, Corpus Christi, USA; 3 Pediatric Critical Care Medicine, Methodist Health System, San Antonio, USA; 4 Biostatistics and Epidemiology, Baylor College of Medicine, Houston, USA; 5 Pediatrics, Texas A&M College of Medicine, College Station, USA; 6 Anesthesiology and Critical Care, University of Texas Medical Branch at Galveston, Galveston, USA; 7 Anesthesiology and Critical Care, Driscoll Children's Hospital, Corpus Christi, USA

**Keywords:** children, fluid assessment, fluid status, pediatric critical care, score

## Abstract

Introduction

Monitoring fluid balance (FB) is important yet challenging in the pediatric intensive care unit (PICU). Currently used, conventional methods of fluid assessment are either not feasible or unreliable. Also, there is a lack of composite scores or clinical algorithms that use different parameters of fluid status. We aim to develop a fluid assessment scoring tool (FAST) to clinically assess the fluid status of children in the PICU.

Methods* *

In this pilot study, we included critically ill patients aged 0-21 years who presented to our PICU between December 2020 and March 2021 and required invasive or non-invasive mechanical ventilation, inotropes, diuretics, and renal replacement therapy. In our study, 32 patients contributed 118 encounters. We assigned scores 0-3 each for fontanelles, eyes, liver exam, daily weight changes, FB, urine output, blood urea nitrogen, creatinine, and chest X-ray findings. We also assigned a score of 0-3 for thoracic fluid content (TFC) as measured by the non-invasive ICON^®^ monitor (Osypka Medical GmbH, Berlin, Germany). First, a non-parametric receiver operating curve (ROC) analysis was used to determine potential cut points for the FAST using the TFC as a “gold standard.” Cut points were selected based on a balanced sensitivity vs. specificity. Next, a 10-fold cross-validation area under the curve (AUC) for ROC analysis was performed to estimate the best-performing cut-point.

Results

The median (interquartile range, IQR) age was 78.7 (9.75-84) months, and 62.5% of patients were males. The median (IQR) FAST score was 4 (2-6). The median (IQR) TFC was 44.5 (29-57.25). Of the 118 encounters, 78 (66%) exhibited high TFC. The two potential FAST scores predicting a higher TFC that indicates fluid overload (FO) were ≥3 (sensitivity=92%; specificity=68%) and ≥4 (sensitivity=80%; specificity=80%). On cross-validation, FAST≥3 (AUC=0.79) and FAST≥4 (AUC=0.78) showed similar performance. When using our FAST cut points to evaluate outcomes, we do not observe significant differences in the median ventilator days, PICU days, or hospital stay days.

Conclusion* *

The FAST offers a practical approach to evaluating FO in critically ill pediatric patients. A FAST score ≥3 was associated with TFC-defined FO, suggesting clinical utility. Future multicenter studies are needed to validate its use and explore its impact on patient outcomes.

## Introduction

Fluid overload (FO) is associated with poor outcomes in critically ill patients [1-6]. FO has been linked to increased 60-day mortality in patients with acute renal failure [3], and the likelihood of cardiopulmonary complications in adult critical care patients rises with the degree of FO [7,8]. In postoperative pediatric cardiac patients, FO is an independent predictor of adverse outcomes [9-13]. Therefore, monitoring fluid balance (FB) is crucial in a pediatric intensive care unit (PICU) setting [14]. However, accurately measuring FB at the bedside in the PICU is challenging. Conventional methods, such as cumulative FB charts and body weight (BW) measurements, often prove to be unreliable or infeasible. For example, a prospective study found that a large proportion of nurse-registered FBs were inaccurate and did not align with standard BW measurements [15]. Similarly, daily weight measurement is often difficult to perform consistently in critically ill children [16]. In a study of 21 children after bone marrow transplant, BW gain and FB demonstrated highly variable sensitivity in detecting FO over 10% [17]. On the other hand, more objective measures, such as thoracic fluid content (TFC) derived from impedance cardiography, have shown promise in reliably assessing FB in pediatric patients [18-21]. A study by Sumbel et al. showed that initial and peak TFC predicted outcomes in critically ill children [22]. Despite this, the limited availability and specialized equipment required for TFC monitoring restrict its widespread clinical use.

Currently, there is no validated composite scoring system that integrates multiple indicators of fluid status to aid clinical decision-making. Most existing methods assess a single parameter in isolation, such as FB, weight changes, or laboratory values, which can be misleading when used independently. Given these limitations, there is a need for a more holistic, feasible, and objective tool to assess FO. 

To address this gap, we hypothesized that the fluid assessment scoring tool (FAST) score would correlate with TFC and serve as a practical screening tool to guide fluid management in the PICU. Our study aimed to assess the feasibility of developing and implementing the FAST, a novel bedside scoring system for critically ill pediatric patients. This tool combines clinical examination findings, laboratory parameters, and imaging results to provide a composite measure of fluid status. This article was previously presented as a meeting abstract at the Society of Critical Care Medicine Congress, February 2022.

## Materials and methods

We conducted this prospective observational study at the Children’s Hospital of San Antonio (CHofSA), a freestanding 200-bed tertiary care children’s hospital. The study was approved by the Baylor College of Medicine Institutional Review Board and the CHofSA Feasibility Committee (H-47900). Given its observational nature, the study was approved with a waiver of informed consent.

We included patients aged 0-21 years admitted to the PICU with acute respiratory failure, shock, or acute kidney injury requiring invasive or non-invasive mechanical ventilation, inotropes, diuretics, and renal replacement therapy. Patients not admitted to the PICU, those admitted under observation status, and those with incomplete data for scoring were excluded.

Our team developed the FAST based on conventional methods of fluid status assessment, including clinical examination, laboratory values, and imaging findings. Each variable was assigned a score from 0 to 3. Parameters included fontanelle status, eye examination, liver size, daily weight changes, FB, urine output, blood urea nitrogen, urine specific gravity, presence of edema, and chest X-ray findings. Table 1 illustrates the FAST developed and used by the study team. 

**Table 1:** Fluid assessment scoring tool The table depicts the scoring tool used to assess fluid overload. BUN, Blood urea nitrogen

Variable	Normal (0 points)	Mild (1 point)	Moderate (2 points)	Severe (3 points)
BUN trend change	Decreased <15%	Decreased 15% to 25%	Decreased 26% to 50%	Decreased >50%
Urine specific gravity	1.010	1.005 to 1.009	1.000 to 1.004	<1.000
Fluid balance change	0 to 15 mL/kg/day	15 to 30 mL/kg/day	30 to 50 mL/kg/day	>50 mL/kg/day
Weight change	<7% increased	Increased by 7%-10%	Increased by 11%-15%	Increased >15%
Urine output (not on diuretics or renal failure)	1 to 3 mL/kg/h	3.1 to 3.5 mL/kg/h	3.51 to 4 mL/kg/h	>4 mL/kg/h
Fontanelle	Flat	Full	Bulging	Bulging and tense
Eyes	Normal appearance	Swollen	Puffy/conjunctival edema	Puffy/unable to open eyes
Liver examination	0 to 0.99 cm below the costal margin	1 to 2 cm below the costal margin	2 to 3 cm below the costal margin	>3 cm below the costal margin
Skin/extremities	No edema	Edema in the limbs or abdomen	Edema in the limbs and face	Anasarca
Chest X-ray (or imaging)	No excessive fluid	Mild pulmonary edema/pleural effusion	Moderate pulmonary edema/pleural effusion	Severe pulmonary edema/pleural effusion

In instances where one or more parameters were unmeasurable (e.g., absence of fontanelles in older children), those variables were omitted. Patients were excluded if more than two variables were missing. Additional parameters such as fluid boluses, blood product administration, and dialysis were reviewed for overall clinical context but were not included in the scoring analysis.

Unlike dehydration, for which established clinical criteria exist, FO lacks well-defined variables and thresholds. Thus, we relied on holistic clinical judgment drawn from daily practice. Chest X-rays were reviewed by a blinded radiologist to reduce interpretive bias.

TFC was also assessed using the non-invasive ICON® monitor (Osypka Medical GmbH, Berlin, Germany), which employs Electrical Cardiometry™ (Osypka Medical GmbH, Berlin, Germany). Four skin sensors were applied to the neck and thorax to measure thoracic conductivity changes. These readings provided a TFC score, categorized as normal, mild, moderate, or severe. Given the lack of a universally accepted gold standard for fluid status in critically ill children, we used TFC as a comparative reference, not as a component of the FAST score. The FAST tool was designed to be filled out by bedside providers, such as nurses or residents, similar to other clinical scoring systems (e.g., Cornell Assessment of Pediatric Delirium and State Behavioral Scale) and aimed to reduce variability by incorporating multiple clinical indicators rather than relying on a single variable such as FB or BW.

We discussed validating the scoring tool using expert opinion, but we were concerned about inaccuracies in differentiating severity categories and thus bias. We collected data across 118 clinical encounters from critically ill PICU patients meeting inclusion criteria. To ensure consistency, the PICU team received structured training on score application. After a one-month familiarization period, routine scoring was initiated and incorporated into daily care. We evaluated patient outcomes from the time of score implementation, focusing on standard critical care metrics: mortality, multiorgan dysfunction, ventilator days, PICU length of stay, and total hospital length of stay.

Sample size

This pilot study included 32 patients who contributed a total of 118 scoring encounters, supporting the initial development and refinement of the FAST for FO assessment in critically ill pediatric patients. As this was an exploratory, hypothesis-generating study focused on tool development rather than hypothesis testing, a formal power calculation was not performed. The selected sample size aligns with commonly accepted ranges for pilot studies and tool development projects, where 30 to 40 participants are often recommended to explore feasibility, estimate variability, and identify preliminary trends. The insights gained from this cohort will inform the design of future studies with larger, adequately powered samples to validate the reliability, clinical utility, and predictive performance of FAST.

Statistical analysis

Descriptive statistics (median with interquartile range (IQR) and mean with standard deviation for continuous variables; proportions for categorical variables) were used to summarize the demographic and clinical characteristics of the patients included in this project, and the clinical characteristics across all events were included in the analysis. No data on key variables were missing for the patients included in this analysis. Due to the sample size of the project, comparisons between groups for continuous variables were assessed using the non-parametric Mann-Whitney U test. No comparisons between groups were made for any categorical variables. To examine the ability of the FAST to classify patients, a non-parametric receiver operating curve (ROC) analysis (roctab procedure in Stata v17.0) was used to determine potential cut points (0-8) for the FAST using the TFC as a “gold-standard.” Cut points were selected based on a balanced sensitivity vs. specificity, and the percentage of patients was correctly classified using that cut-point. Next, a 10-fold cross-validation area under the curve (AUC) for ROC analysis was performed to estimate the best-performing cut-point. To understand the association between the top two FAST score cut points and clinical outcomes (i.e, ventilator, PICU, and hospital days), we compared the median number of days for each outcome between those below and above the two best cut points. p-values were calculated using the Mann-Whitney U test for group comparisons. A p-value ≤0.05 was considered statistically significant.

## Results

**Figure 1:**
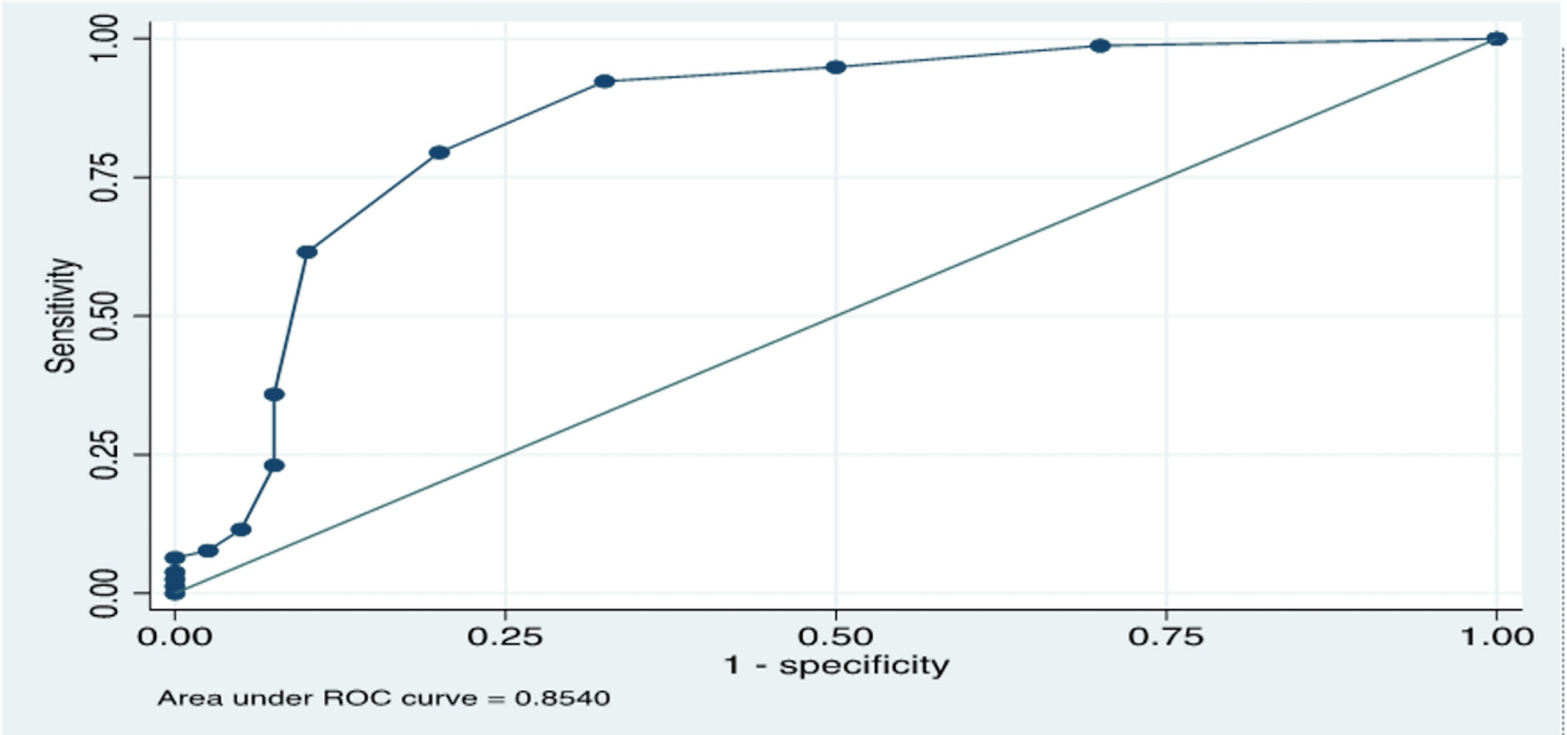
ROC curve for fluid assessment scoring tool ROC, Receiving operating curve

Two candidate FAST score cut points were evaluated against TFC using ROC analysis: a score of ≥3 yielded a sensitivity of 92% (95% CI: 84%-97%) and specificity of 68% (95% CI: 51%-81%), with 83.9% of patients correctly classified. A score of ≥4 had a sensitivity of 79% (95% CI: 69%-88%) and a specificity of 80% (95% CI: 64%-91%), correctly classifying 79.7% of patients (Table 2). Cross-validation analysis showed a slightly higher AUC for the FAST ≥3 model (AUC=0.79; 95% CI: 0.72-0.88) than the FAST ≥4 (AUC=0.78; 95% CI: 0.72-0.87). The superior sensitivity (92.3% vs. 79.5%) and higher overall classification accuracy (83.9% vs. 79.7%) further support the FAST ≥3 threshold as the optimal cut-point for identifying FO in this population (Figure 1).

**Table 2:** Cut-point determination for fluid overload score

Cut point	Sensitivity	Specificity	Correctly classified
≥0	100.00%	0.00%	66.10%
≥1	98.72%	30.00%	75.42%
≥2	94.87%	50.00%	79.66%
≥3	92.31%	67.50%	83.90%
≥4	79.49%	80.00%	79.66%
≥5	61.54%	90.00%	71.19%
≥6	35.90%	92.50%	55.08%
≥7	23.08%	92.50%	46.61%
≥8	11.54%	95.00%	39.83%

Despite stratifying patients by FAST cut points, no statistically significant differences were observed in critical care outcomes, including ventilator days, PICU length of stay, or total hospital stay, across the score thresholds (Table 3).

**Table 3:** Association of FOS cut points and clinical outcomes p-values were calculated using the Mann-Whitney U test for group comparisons. A p-value ≤0.05 was considered statistically significant. FAST, Fluid assessment scoring tool; FOS, Fluid overload score; IQR, Interquartile range; PICU, Pediatric intensive care unit

Clinical outcomes/FAST	FOS<3	FOS≥3	X^2^	p-value		FOS<4	FOS≥4	X^2^	p-value
Ventilator days (median (IQR))	11 (5-23)	8.5 (3-22)	0.8374	0.36		11 (4-24)	8 (3-20)	2.343	0.13
PICU days (median [IQR])	18 (6-31)	14 (4-24.5)	1.0482	0.31		17.5 (6-34)	14 (4-22)	1.6741	0.20
Hospital days (median [IQR])	20 (6-33)	15 (6-28)	0.5396	0.46		19.5 (7-35)	14.5 (5-25.5)	0.8285	0.36

These results demonstrate the potential of the FAST score, particularly the FAST ≥3 threshold, to correlate with FO status as measured by TFC, warranting further validation in larger, powered studies.

## Discussion

Our study attempted to develop a novel FAST for critically ill children. Using clinical and laboratory parameters, the scoring tool, FAST, was able to demonstrate a cut point to evaluate the fluid status in critically ill children. To our knowledge, this is one of the first scoring tools designed to objectively assess FO in pediatric critical care using a multidomain clinical approach. Similar to other scoring tools used in the PICU, the FAST score could be integrated into bedside assessments by nurses or front-line providers. PICU patients are assessed by nurses throughout the day, with parameters entered and scores measured. 

Multiple studies have demonstrated an association between FO and poor outcomes in critically ill patients [1-6]. An increasing degree of FO has been associated with an increased likelihood of cardiopulmonary complications in adult critical care patients. Less fluid gain and lower lung water are associated with more ventilator-free days and shorter intensive care unit (ICU) and hospital length of stays [7,8]. Arikan and co-authors showed that FO may adversely affect the prognosis of children who did not receive continuous renal replacement therapy [23]. Unfortunately, the best method for measuring FO at the bedside in the PICU is unclear. Most of the aforementioned studies used FB as the sole measure of FO. Our team developed a FAST that helps determine the fluid status of the patient, taking into consideration the different variables used to assess pediatric patients’ fluid status in the PICU. The study does not refute the validity of the variables used, but it does indeed rectify the flaws that are encountered when using those variables. This is why we need to take all variables (or at least most of them, when possible) to better assess the fluid status of a particular patient. This method is not perfect, but it does cover most of the clinical variables used daily. When using most of the variables discussed, the bias in assessing FB will decrease to a minimum, and the patient will have an appropriate measure of the fluid status.

Conventional methods

FB vs. BW

While FB is one of the variables that is used to assess the overall FO of the patient, if not considered in the context of other variables (BW and physical examination), it could be misleading. To reduce confusion around terminology, the definitions of FB and FO were addressed in detail in a report from the Pediatric Acute Disease Quality Initiative conference [24]. The report recommends using the term FO to describe a pathological state of positive FB associated with adverse clinical outcomes [24]. The FO and Kidney Injury Score (FOKIS) is a multidimensional scoring system developed to stratify risks associated with kidney injury, FO, and nephrotoxic medication exposure in children admitted to the ICU [25]. However, this tool is primarily a measure of kidney injury burden, not a bedside decision-making guide for managing fluid intake or diuretic therapy. Additionally, FOKIS defines FO as the ratio of "total fluid in" minus "total fluid out" to ICU admission weight. This approach does not take into consideration the clinical impact or pathological significance of those fluid values in individual patients [25]. In a prospective study on the assessment of FB in critically ill patients, a large proportion of nurse-registered cumulative FBs were inaccurate and did not agree with standard BW measurements [15]. Although daily BW measurement in critically ill patients is a good indicator of the day-to-day fluid status, it has been found difficult to perform consistently, even with electronic beds and highly trained nurses. When compared with the results of the FB, it was weakly correlated and had a progressively greater difference with greater changes in BW [16]. In this study, the investigators measured compliance with BW measurement. They found that compliance decreased during the study period. The reasons for non-compliance with the protocol were failure to calibrate the bed before admission or failure to complete the procedure. Similar results were demonstrated in a study of 21 children admitted to the PICU after bone marrow transplantation, where BW and FB have shown a highly variable sensitivity to detect a FO of over 10% [17]. Another study assessed the accuracy of the FB by comparing it with the changes in BW in 32 patients undergoing cardiac surgery. The BW was obtained before the operation and on ICU discharge. They found that only 9.75% of the cases had a correct estimated change in BW compared with FB. The BW changes were underestimated in over half of the patients and overestimated in close to a third of the patients. In 12% of cases, there was a difference greater than a liter [26].

Chest Radiographs

Chest X-rays are one of the most frequently used diagnostic methods in the PICU. Signs of volume overload include dilated upper lobe vessels, cardiomegaly, interstitial edema, enlarged pulmonary artery, pleural effusion, alveolar edema, prominent superior vena cava, and Kerley lines. Fluid retention in the lungs could be related to local cardiopulmonary and lymphatic abnormalities, such as pneumonia, lymphatic malformation, and pulmonary vein stenosis or other systemic abnormalities. According to one study, one in five adult patients admitted from the ED with acute decompensated heart failure had no signs of congestion on chest radiography [27]. Moreover, most of the Chest X-rays taken in the PICU are portable, which reduces the sensitivity of findings of volume overload [28]. Thus, chest X-ray is not a reliable method for measuring the overall fluid status of the patient. It can, however, be used with other parameters to assess FO.

Non-conventional methods

Ultrasonography

Ultrasound can be used to assess the thoracic fluid burden, assessing for pleural effusion, pericardial effusion, or B-lines that are suggestive of FO. Picano et al. concluded that the ultrasound lung comets sign is a good way to directly image extravascular lung water [29]. Moreover, inferior vena cava diameter measurement can also be used for the prediction of the overall fluid status of adult patients. This method becomes difficult to apply to critically ill children, given the hemodynamic changes that happen when pediatric patients are intubated. For this reason, it was suggested to use the collapsibility and distensibility indices to better assess the fluid status of critically ill pediatric patients [30]. Although this is a non-invasive, radiation-free method to measure fluid status, it continues to require training and experience to decrease the subjectivity in measurements made by different providers.

TFC

Objective measures of FB, such as TFC, have been found to be more reliable in assessing FB in critically ill children [18-21]. A study conducted by our team showed that initial and peak TFC predicted outcomes in critically ill children [22]. However, the use of TFC is not widely available, and a more convenient way of measuring the fluid status of children in the ICU is needed.

There is an increasing need for an objective way to measure FO in pediatric patients in the PICU. Our team developed the FAST as an objective measurement of the overall fluid status of children in the PICU. This tool uses daily examinations and laboratory results to assess the fluid status of children in the PICU. We suggest applying the tool to patients once a shift. It can be performed by the nurse or front-line provider on select patients. It can also be incorporated into the daily assessment of the patient. This tool can reflect the overall fluid status of the patient and serve as a guide for fluid management. It is not a substitute for a comprehensive physical examination and thorough assessment by a healthcare provider.

Limitations

Our study demonstrated a correlation between a higher FAST score and FO as measured by TFC. This study examines the feasibility of developing a scoring tool to avoid interindividual variability and bias. For this reason, multiple variables are used. While using this scoring tool, or a modified version of it, the disagreements among individuals will be reduced to a minimum since we are looking at the whole picture instead of one variable (e.g., FB). Also, some of these variables are not measured daily due to multiple reasons, including the severity of illness or the unavailability of electronic beds. In this situation, the score of the other variables will reflect the overall fluid status. For example, obtaining the daily weight of intubated patients is not feasible, and the score accumulated by other variables will be sufficient to adjust for the lost variable. However, a larger sample size would have given more power to the study and made it more generalizable. This could be why we were unable to demonstrate statistical significance with the measured outcomes. We were also unable to find a “gold standard” in the literature. For this reason, we had to use TFC measured by the ICON^®^ monitor as a “gold standard.” This could be avoided by blinding two or more PICU physicians from the scoring tool and using their examination as the "gold standard". This study presents the initial development and findings of a scoring tool designed to assess FO, which should be validated through future research. We emphasize the need for external validation of this tool before integration into the clinical workflow. 

## Conclusions

The FAST could be the platform for the development of a robust tool for clinically measuring the overall fluid status and FO of children in the PICU. This tool uses daily examinations and laboratory results to assess the fluid status of children in the PICU. A FAST score ≥3 was associated with TFC-defined FO. While this pilot study did not demonstrate statistically significant outcome differences, trends suggest that higher scores may correlate with worse clinical outcomes. Future multicenter studies are needed to validate these findings and refine the scoring system.
